# Thiazide Diuretics and the Incidence of Osteoporotic Fracture: A Systematic Review and Meta-Analysis of Cohort Studies

**DOI:** 10.3389/fphar.2019.01364

**Published:** 2019-11-21

**Authors:** Jun Wang, Ke Su, Weihua Sang, Longjie Li, Shiyun Ma

**Affiliations:** The Second Department of Orthopedics, Cangzhou Central Hospital, Cangzhou, China

**Keywords:** thiazide diuretics, osteoporotic fracture, osteoporosis, cohort study, meta-analysis, systematic review

## Abstract

**Background:** Thiazide diuretics may improve bone mineral density. However, results are inconsistent for studies evaluating the association between thiazides and risk of osteoporotic fracture. We performed an updated meta-analysis of cohort studies to determine the association between thiazides use and fracture risk.

**Methods:** Relevant studies were identified *via* systematic search of PubMed and Embase. A random-effect model was used for meta-analysis. Subgroup analyses were performed to explore the potential influences of study characteristics on the outcome.

**Results:** Seventeen cohort studies with 3,537,504 participants were included. The pooled results showed that use of thiazide diuretics at baseline did not significantly affect the risk of overall osteoporotic fracture incidence as compared with controls (risk ratio [RR]: 0.96, 95% confidence interval [CI]: 0.83 to 1.09, p = 0.51) with significant heterogeneity (p for Cochrane’s Q test < 0.001, I^2^ = 90%). Results of subgroup analyses indicated that general status of the participants may be an important determinant for the association between thiazide diuretics and subsequent risk of osteoporotic fracture. Use of thiazide diuretics was associated with significantly reduced risk of fracture in patients with acute status including new-onset stroke or spinal cord injury (RR: 0.70, 95% CI: 0.57 to 0.86, p < 0.001), but not in those with good conditions such as community-dwelling population or hypertensive patients (p for subgroup difference = 0.02).

**Conclusions:** Use of thiazide diuretics is not associated with significantly affected risk of overall osteoporotic fracture. However, the association may be different according to the general status of the participants.

## Introduction

Osteoporosis is a common skeletal disease characterized by increased bone fragility and risk of fracture (Sozen et al., 2017; Figliomeni et al., 2018). As an important cause of morbidity and mortality in people over 65 years, osteoporotic fracture leads to pain and loss of functional ability to these patients, and medical costs for the prevention and treatment of fractures have become substantial for both the developed and the developing countries (Oden et al., 2015). Therefore, the identification of factors that may affect the development of osteoporotic fracture is of significance for the prevention and treatment of the disease. Conventionally, many factors have been considered to potentially increase the risk of osteoporotic fracture, including aging, low bone mineral density (BMD), obesity, comorbidities of diabetes and vascular diseases, as well as poor dietary habits with excessive alcohol consumption, and low supplements of calcium and vitamins (Drake et al., 2012).

Besides, some medications that influence the calcium homeostasis of the body are also suggested to affect the risk of osteoporotic fracture. Thiazide diuretics, one of the most commonly prescribed categories of medications for patients with hypertension and other cardiovascular diseases (Olde Engberink et al., 2015), have been suggested to favorably affect BMD *via* their efficacy for lowering the urinary excretion of calcium (Sigurdsson and Franzson, 2001; Alexander and Dimke, 2017). Indeed, early observational studies have demonstrated that users of thiazide diuretics are associated with greater BMD as compared with nonusers (Laroche and Mazieres, 1998; Sigurdsson and Franzson, 2001). Moreover, these findings were further confirmed by results of randomized controlled trials (RCTs) which showed that thiazide diuretics are associated with better preserved BMD as compared with placebo in high-risk people of osteoporosis, such as participants over 65 and postmenopausal women (Transbol et al., 1982; Wasnich et al., 1995; Lacroix et al., 2000). Therefore, it was hypothesized that the benefits of thiazide diuretics on BMD may translate to the prevention of osteoporotic fractures. Subsequently, many observational studies have been published to evaluate the potential association between use of thiazide diuretics and the risk of osteoporotic fracture (Lacroix et al., 1990; Cauley et al., 1993; Nguyen et al., 1996; Feskanich et al., 1997; Guo et al., 1998; Schoofs et al., 2003; Meisinger et al., 2007; Solomon et al., 2011; Butt et al., 2012; Carbone et al., 2014; Ruths et al., 2015; Torstensson et al., 2015; Chen et al., 2016; Paik et al., 2016; Bokrantz et al., 2017; Hargrove et al., 2017; Lin et al., 2017). Although the findings of these studies were inconsistent, previous meta-analyses by including these studies generally indicated that use of thiazide diuretics are associated with about 20% reduced risk of fracture events, mainly driven by studies with primary outcomes of hip fractures (Jones et al., 1995; Wiens et al., 2006; Aung and Htay, 2011; Xiao et al., 2018). However, both case-control and cohort studies were included in these meta-analyses and including of case-control studies may introduce additional bias (Austin et al., 2012). Moreover, substantial recently published cohort studies were not included in previous meta-analyses (Solomon et al., 2011; Butt et al., 2012; Carbone et al., 2014; Ruths et al., 2015; Torstensson et al., 2015; Chen et al., 2016; Paik et al., 2016; Bokrantz et al., 2017; Hargrove et al., 2017; Lin et al., 2017), and an updated meta-analysis is therefore needed to summarize current understanding of the association between thiazide diuretics and risk of osteoporotic fracture. In addition, although the optimal strategy to evaluate the above hypothesis is to perform RCTs, due to the potential moderate effect of thiazide diuretics on fracture risk, these RCTs remain unavailable because too many high-risk osteoporotic participants are needed to make the studies of adequate power to detect the potential efficacy of thiazides (Jones et al., 1995). Therefore, in this study, we performed an updated meta-analysis of cohort studies to systematically evaluate the potential association between use of thiazide diuretics and the risk of overall osteoporotic fracture incidence.

## Materials and Methods

The meta-analysis was performed in accordance with the MOOSE (meta-analysis of observational studies in epidemiology) (Stroup et al., 2000) and Cochrane’s Handbook (Higgins and Green, 2011) guidelines.

### Literature Search

Databases of PubMed and Embase were searched from the index date for relevant records, using the combinations of the following terms (1) “diuretic” OR “diuretics” OR “thiazide” OR “thiazides” OR “hydrochlorothiazide” OR “chlorthalidone”, “bendroflumethiazide” OR “chlorothiazide” OR “cyclothiazide” OR “methyclothiazide” OR “hydroflumethiazide” OR “trichlormethiazide” OR “benzthiazide” OR “polythiazide” OR “buthiazide” OR “cyclopenthiazide” OR “metolazone” OR “quinethazone” OR “fenquizone” OR “clorexolone” OR “clopamide” OR “indapamide” OR “diapamide” OR “isoindapamide” OR “mefruside” OR “xipamide”; AND (2) “fracture”; AND (3) “prospective” OR “prospectively” OR “retrospective” OR “retrospectively” OR “followed” OR “follow-up” OR “cohort” OR “cohorts”. The search was limited to studies in humans and published in English language. The reference lists of original and review articles were also analyzed using a manual approach. The final literature search was performed on February 26, 2019.

### Study Selection

Articles were included in the meta-analysis if they met all of the following criteria: (1) published as full-length article in English; (2) reported as cohort studies (prospective or retrospective, regardless of sample size or the follow-up duration); (3) included adult population (≥ 18 years of age) without fracture at baseline; (4) use of thiazide diuretics were defined as exposure of interest at baseline, while participants that did not use thiazide diuretics were defined as controls; (5) documented the incidence of any osteoporotic fracture during follow-up; and (6) reported the risk RRs and their corresponding 95% CIs for the incidence of osteoporotic fracture comparing participants that used thiazide diuretics at baseline to those did not use thiazide diuretics. Reviews, letters, editorials, nonhuman studies, and studies with designs other than cohort study were excluded.

### Data Extracting and Quality Evaluation

Literature search, data extraction, and quality assessment of the included studies were performed independently by two reviewers (J.W. and K.S.) according to the predefined inclusion criteria. Discrepancies were resolved by consensus. Data that were extracted include: (1) name of first author, publication year, and country where the study was performed; (2) design characteristics (prospective or retrospective); (3) sources, characteristics, and numbers of the participants; (4) strategies to confirm the using of thiazide diuretics at baseline; (5) enrollment year and follow-up durations; (6) definitions of fracture outcomes, number of cases with osteoporotic fracture, and strategies to confirm facture outcome during follow-up; and (7) variables adjusted when presenting the results. The quality of each study was evaluated using the Newcastle-Ottawa Scale (Wells et al., 2010) which ranges from 1 to 9 stars and judges each study regarding three aspects: the selection of the study groups; the comparability of the groups; and the ascertainment of the outcome of interest.

### Statistical Analyses

We used RRs as the general measure for the association between use of thiazide diuretics at baseline and the incidence of fracture. Data of RRs and their corresponding standard errors (SEs) were calculated from 95% CIs or p values, and were logarithmically transformed to stabilize variance and normalized the distribution (Higgins and Green, 2011). The Cochrane’s Q test and I^2^ test were used to evaluate the heterogeneity among the included cohort studies (Higgins and Thompson, 2002). A significant heterogeneity was considered if I^2^ > 50%. We used a random-effect model to synthesize the RR data because this model is considered as a more generalized method which incorporates of the potential heterogeneity (Higgins and Green, 2011). Sensitivity analyses, by removing individual study one at a time, were performed to test the robustness of the results (Patsopoulos et al., 2008). Predefined subgroup analyses were performed to evaluate whether the association between the use of thiazide diuretics at baseline and the incidence of fracture was affected by study design characteristics, sex of the participants, number of the participants, population characteristics, follow-up durations, site of fractures, strategies to confirm use of thiazide diuretics, and the numbers of variables adjusted when presenting the results. Moreover, potential publication bias was assessed by funnel plots with the Egger regression asymmetry test (Egger et al., 1997). We used the RevMan (Version 5.1; Cochrane Collaboration, Oxford, UK) and STATA software for the meta-analysis and statistics.

## Results

### Literature Search

**Figure 1:**
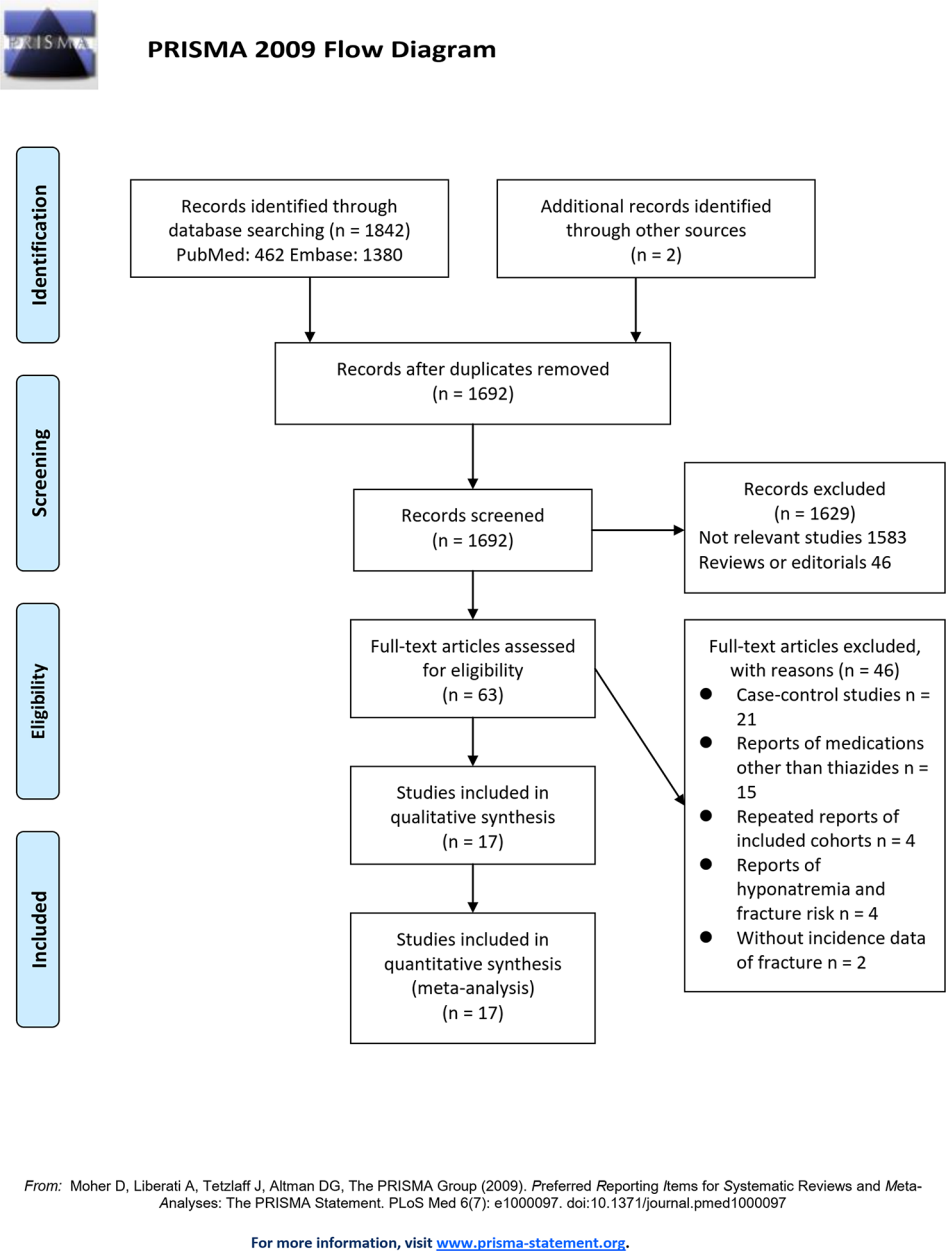
Flowchart of database search and study identification.

The flowchart of database search was presented in **Figure 1**. Briefly, 1,842 articles were found *via* initial literature search of the PubMed and Embase databases, and two studies were found *via* manual search of the reference lists of the review articles. After exclusion of 152 duplications, 1,692 articles underwent screening. Subsequently, 1,629 were excluded through screening of the titles and abstracts mainly because they were not relevant to the purpose of the meta-analysis. Subsequently, 63 potential relevant records underwent full-text review. Of these, 46 were further excluded because 21 of them were case-control studies, 15 were reports of medications other than thiazide diuretics as exposure, four were repeated reports of included cohorts, four were studies evaluating the association between hyponatremia and fracture risk, and the other two did not report the incidence data of fracture outcome. Finally, 17 cohort studies were included (Lacroix et al., 1990; Cauley et al., 1993; Nguyen et al., 1996; Feskanich et al., 1997; Guo et al., 1998; Schoofs et al., 2003; Meisinger et al., 2007; Solomon et al., 2011; Butt et al., 2012; Carbone et al., 2014; Ruths et al., 2015; Torstensson et al., 2015; Chen et al., 2016; Paik et al., 2016; Bokrantz et al., 2017; Hargrove et al., 2017; Lin et al., 2017).

### Study Characteristics and Quality Evaluation

The characteristics of the included studies were summarized in **Tables 1** and **2**. Overall, we included 17 cohort studies (Lacroix et al., 1990; Cauley et al., 1993; Nguyen et al., 1996; Feskanich et al., 1997; Guo et al., 1998; Schoofs et al., 2003; Meisinger et al., 2007; Solomon et al., 2011; Butt et al., 2012; Carbone et al., 2014; Ruths et al., 2015; Torstensson et al., 2015; Chen et al., 2016; Paik et al., 2016; Bokrantz et al., 2017; Hargrove et al., 2017; Lin et al., 2017) with a total of 3,537,504 participants. Eight of the included studies were performed in North America (Lacroix et al., 1990; Cauley et al., 1993; Feskanich et al., 1997; Solomon et al., 2011; Butt et al., 2012; Carbone et al., 2014; Paik et al., 2016; Hargrove et al., 2017), six in Europe (Guo et al., 1998; Schoofs et al., 2003; Meisinger et al., 2007; Ruths et al., 2015; Torstensson et al., 2015; Bokrantz et al., 2017), and the other three in Australia or Asia (Nguyen et al., 1996; Chen et al., 2016; Lin et al., 2017). Nine of them were designed as prospective cohort studies (Lacroix et al., 1990; Cauley et al., 1993; Nguyen et al., 1996; Feskanich et al., 1997; Guo et al., 1998; Schoofs et al., 2003; Meisinger et al., 2007; Solomon et al., 2011; Paik et al., 2016), while the other eight were retrospective cohort studies (Butt et al., 2012; Carbone et al., 2014; Ruths et al., 2015; Torstensson et al., 2015; Chen et al., 2016; Bokrantz et al., 2017; Hargrove et al., 2017; Lin et al., 2017). The sample sizes of the included studies varied between 820 and 1,586,554. Ten studies included community-dwelling general population (Lacroix et al., 1990; Cauley et al., 1993; Nguyen et al., 1996; Feskanich et al., 1997; Guo et al., 1998; Schoofs et al., 2003; Meisinger et al., 2007; Ruths et al., 2015; Torstensson et al., 2015; Paik et al., 2016), five included patients with hypertension (Solomon et al., 2011; Butt et al., 2012; Chen et al., 2016; Bokrantz et al., 2017; Hargrove et al., 2017), while the other two included patients with new-onset stroke or spinal cord injury (Carbone et al., 2014; Lin et al., 2017). Baseline use of thiazide diuretics was confirmed *via* self-report by the patients in seven studies (Lacroix et al., 1990; Cauley et al., 1993; Nguyen et al., 1996; Feskanich et al., 1997; Guo et al., 1998; Meisinger et al., 2007; Paik et al., 2016), while in the other studies, use of thiazide diuretics were confirmed based on the prescription records (Schoofs et al., 2003; Solomon et al., 2011; Butt et al., 2012; Carbone et al., 2014; Ruths et al., 2015; Torstensson et al., 2015; Chen et al., 2016; Bokrantz et al., 2017; Hargrove et al., 2017; Lin et al., 2017). With a mean follow-up between 0.5 to 11 years, 312,246 cases of osteoporotic fracture occurred based on the reports from the patients or general practitioners, or identified from healthcare databases. For the definitions of osteoporotic fracture outcomes, hip fracture was reported in nine studies (Lacroix et al., 1990; Cauley et al., 1993; Feskanich et al., 1997; Guo et al., 1998; Schoofs et al., 2003; Solomon et al., 2011; Butt et al., 2012; Ruths et al., 2015; Lin et al., 2017), while some studies reported the overall incidences of osteoporotic fractures (Cauley et al., 1993; Nguyen et al., 1996; Meisinger et al., 2007; Solomon et al., 2011; Torstensson et al., 2015; Chen et al., 2016; Bokrantz et al., 2017; Hargrove et al., 2017). When presenting the results, potential confounding variables such as age, sex, comorbidities, baseline BMD, and concurrent medications et al. were adjusted variably. The qualities of the included cohorts were generally good, with NOS scores ranging between 6 and 9.

**Table 1:** Baseline characteristics of the included studies.

Study	Country	Design	Participants characteristics	Number of participants	Age	Male	Enrollment year	Thiazide ascertainment
					years	%		
Lacroix et al.,1990	USA	PC	Community-based population ≥ 65 years	9,518	74.2	39	1981∼1983	Self-report
Cauley et al.,1993	USA	PC	Community-based women ≥ 65 years	9,704	71.8	0	1986∼1988	Self-report
Nguyen et al.,1996	Australia	PC	Community-based population ≥ 60 years	820	NA	100	1989	Self-report
Feskanich et al.,1997	USA	PC	Women aged 36 to 61 years	83,728	49.2	0	1982	Self-report
Guo et al.,1998	Sweden	PC	Community-based population ≥ 75 years	1,608	82	NA	1987	Self-report
Schoofs et al., 2003	the Netherlands	PC	Community-based population ≥ 55 years	7,891	68.9	38.9	1990∼1993	Prescriptions from computerized pharmacies
Meisinger et al., 2007	Germany	PC	Community-based population aged 55∼74 years	1,793	62.3	46.5	1984∼1985	Self-report
Solomon et al., 2011	USA	PC	Hypertensive patients ≥ 65 years with single antihypertensive medication	376,061	80.2	19.3	NA	Prescription filling by Medicare
Butt et al., 2012	Canada	RC	Hypertensive patients ≥ 65 years	301,591	80.8	19.3	2000	Prescription drugs database
Carbone et al., 2014	USA	RC	Hospitalized men with spinal cord injury	6,969	58.2	100	2002	Prescription based on a clinical administrative database
Ruths et al., 2015	Norway	RC	Community-based population ≥ 60 years	906,422	72.8	44	2004∼2010	National prescription database
Torstensson et al., 2015	Denmark	RC	Community-based population ≥ 65 years	1,586,554	74.8	47.2	1999∼2012	National prescription database
Paik et al., 2016	USA	PC	Community-based women ≥ 55 years	55,780	66.7	0	2002∼2012	Self-report
Chen et al., 2016	China	RC	Hypertensive patients ≥ 65 years	1,144	75.9	43.6	2002	Prescription database
Lin et al., 2017	China	RC	Hospitalized patients with new-onset ischemic stroke	7,470	NA	57.6	2000∼2011	Prescription database
Hargrove et al., 2017	USA	RC	Hypertensive patients ≥ 65 years	122,629	75	39	2008∼2011	Prescription filling by Medicare
Bokrantz et al., 2017	Sweden	RC	Hypertensive patients ≥ 45 years	57,822	66	45	2001∼2008	Prescribed Drug register

NA, not available; PC, prospective cohort; RC, retrospective cohort.

**Table 2:** Characteristics of follow-up and outcome of the included studies.

Study	Follow-up	Fracture ascertainment	Fracture cases	Fracture sites	Adjusted factors	NOS scores
	years					
Lacroix et al., 1990	3.6	Self-report or medical record confirmed	242	Hip	Age, sex, impaired mobility, BMI, smoking, alcohol consumption, and history of DM	8
Cauley et al., 1993	3.3	Self-report or radiography confirmed	1,113	Osteoporotic fracture (including hip)	Age, weight, functional status, total calcium intake, years of estrogen replacement, self-reported health status, and level of distal radius bone mass	8
Nguyen et al., 1996	5	Radiography confirmed	166	Osteoporotic fracture	Age, BMD	6
Feskanich et al., 1997	9.2	Self-report	1,845	Forearm and hip	Age, follow-up period, BMI, menopausal status, postmenopausal estrogen use, smoking, alcohol drinking, dietary intake of calcium, vitamin D, histories of heart diseases and osteoporosis	8
Guo et al., 1998	4.5	Inpatient register system confirmed	134	Hip	Age, sex, education, institution as residence, limitation of activities of daily living, histories of stroke, tumor, and cognitive impairment	8
Schoofs et al., 2003	7.4	GP-report	281	Hip	Age, sex, lower-limb disability, BMI, estrogen use, and current smoking	8
Meisinger et al., 2007	10.7	Self-report	263	Osteoporotic fracture	None	6
Solomon et al., 2011	0.5	Health care utilization data confirmed	2,543	Osteoporotic fracture (including hip)	Age, sex, race, other medications, comorbidity scores, BMD and histories of osteoporosis	8
Butt et al., 2012	10	Health care database confirmed	1,463	Hip	Age, sex	7
Carbone et al., 2014	5	Health care registry confirmed	832	Hip	Age, race, severity of spinal cord injury, Charlson comorbidity index, history of seizers, and concurrent medications	8
Ruths et al., 2015	5.2	National fracture registry confirmed	39,938	Hip	Age, sex	6
Torstensson et al., 2015	6.7	National fracture registry confirmed	255,936	Osteoporotic fracture	Age, sex, calendar year, comorbidities, and exposure to the other classes of CVD-drugs	8
Paik et al., 2016	9.7	Self-report and medical record or radiography confirmed	420	Vertebral	BMI, race, physical activity, history of falls, smoking status, alcohol intake, supplemental calcium intake, quintiles of diet calcium intake, total vitamin D intake, vitamin A intake, total protein intake, self-reported diabetes or osteoporosis, history of beta-blocker use, bisphosphonate use, oral steroid use, or postmenopausal hormone use, and recent physical examination	9
Chen et al., 2016	11	Health care registry confirmed	128	Osteoporotic fracture	Age, sex, comorbidities, and concurrent medication	7
Lin et al., 2017	2	Health care registry confirmed	167	Hip	Age, sex, socioeconomic factors, stroke severity, comorbidities, and concurrent medication	7
Hargrove et al., 2017	1	Health care utilization data confirmed	4,430	Osteoporotic fracture	Age, sex, frailty index, socioeconomic factors, comorbidities, and concurrent medication	8
Bokrantz et al., 2017	6	Health care utilization data confirmed	2,345	Osteoporotic fracture	Age, sex, previous fracture, smoking, diabetes mellitus, cerebrovascular disease, chronic obstructive pulmonary disease, Parkinson’s disease, alcoholism, use of antihypertensives other than thiazides (separately by each drug class), antiosteoporotic treatment, glucocorticosteroids, antidepressants/anxiolytics/sedatives, neuroleptics, antiepileptics, hormone replacement therapy, ethnicity, and educational level	8

BMD, bone mineral density; BMI, body mass index; CVD, cardiovascular diseases; DM, diabetes mellitus; GP, general practitioner.

### Association Between Use of Thiazide Diuretics At Baseline and Fracture Incidence

**Figure 2:**
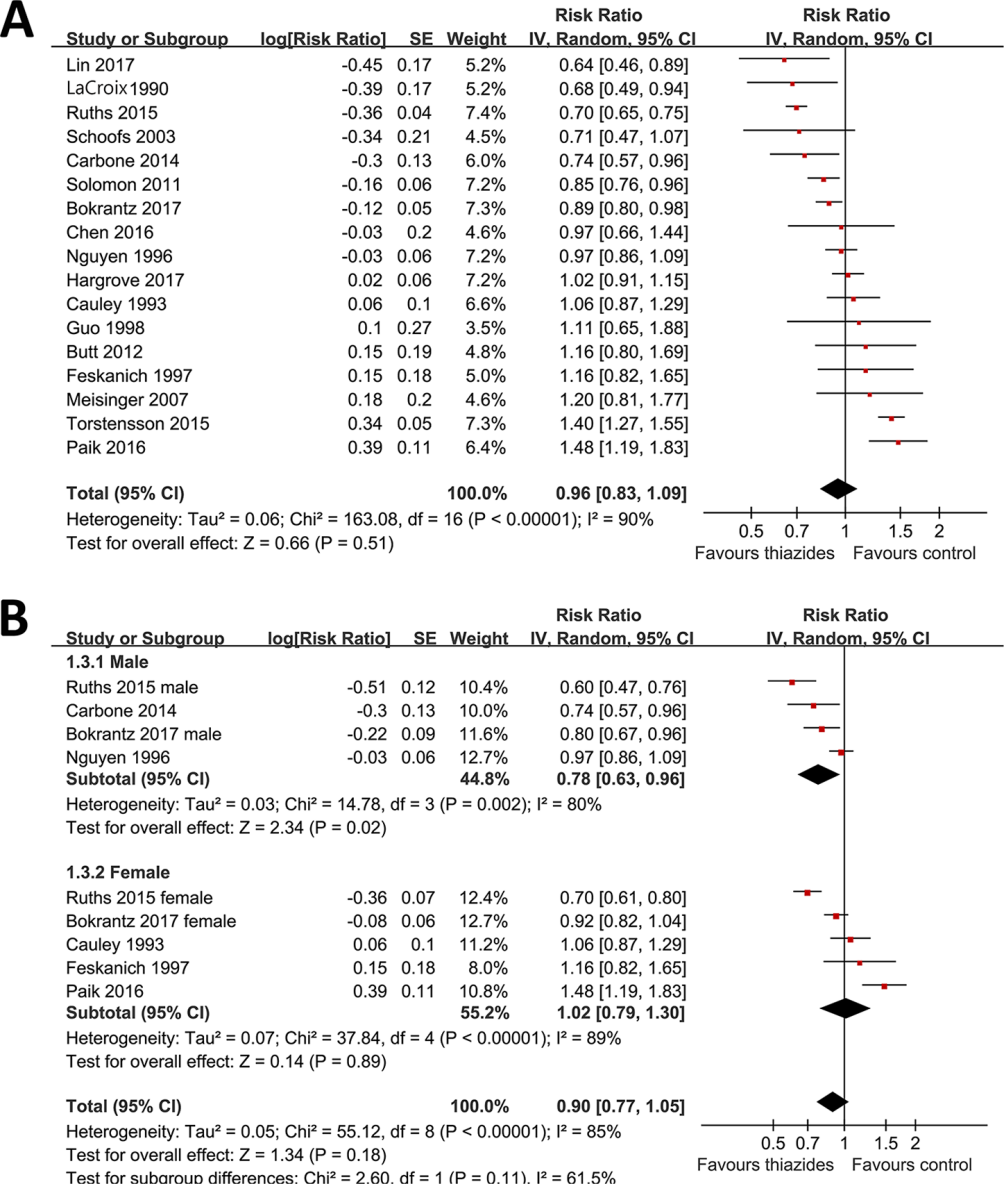
Forest plots for the meta-analysis of the association between the use of thiazide diuretics and the incidence of fracture. **(A)** Forest plots for the overall participants; **(B)** forest plots for the subgroup analysis by sex.

Using a randomized-effect model, the pooled results of data from 17 cohorts indicated that use of thiazide diuretics at baseline did not significantly affect the risk of overall osteoporotic fracture incidence as compared with controls (RR: 0.96, 95% CI: 0.83 to 1.09, p = 0.51; **Figure 2A**) with significant heterogeneity (p for Cochrane’s Q test < 0.001, I^2^ = 90%). Results of sensitivity analyses by omitting one study at a time did not significantly change the results (RR: 0.92∼0.98, p: 0.17∼0.77). Two of the studies included male participants exclusively (Nguyen et al., 1996; Carbone et al., 2014), three included female participants exclusively (Cauley et al., 1993; Feskanich et al., 1997; Paik et al., 2016), and two studies reported the outcome in male and female participants separately (Ruths et al., 2015; Bokrantz et al., 2017). By pooling the data from above studies, we found that use of thiazide diuretics at baseline was associated with significantly reduced incidence of overall osteoporotic fracture in male participants (RR: 0.78, 95% CI: 0.63 to 0.96, p = 0.02; I^2^ = 80%), but not in female participants (RR: 1.02, 95% CI: 0.79 to 1.30, p = 0.89; I^2^ = 89%; **Figure 2B**). However, the results between the subgroups by sex were not significantly different (p = 0.11 for subgroup differences).

### Results of Other Subgroup Analyses

**Figure 3:**
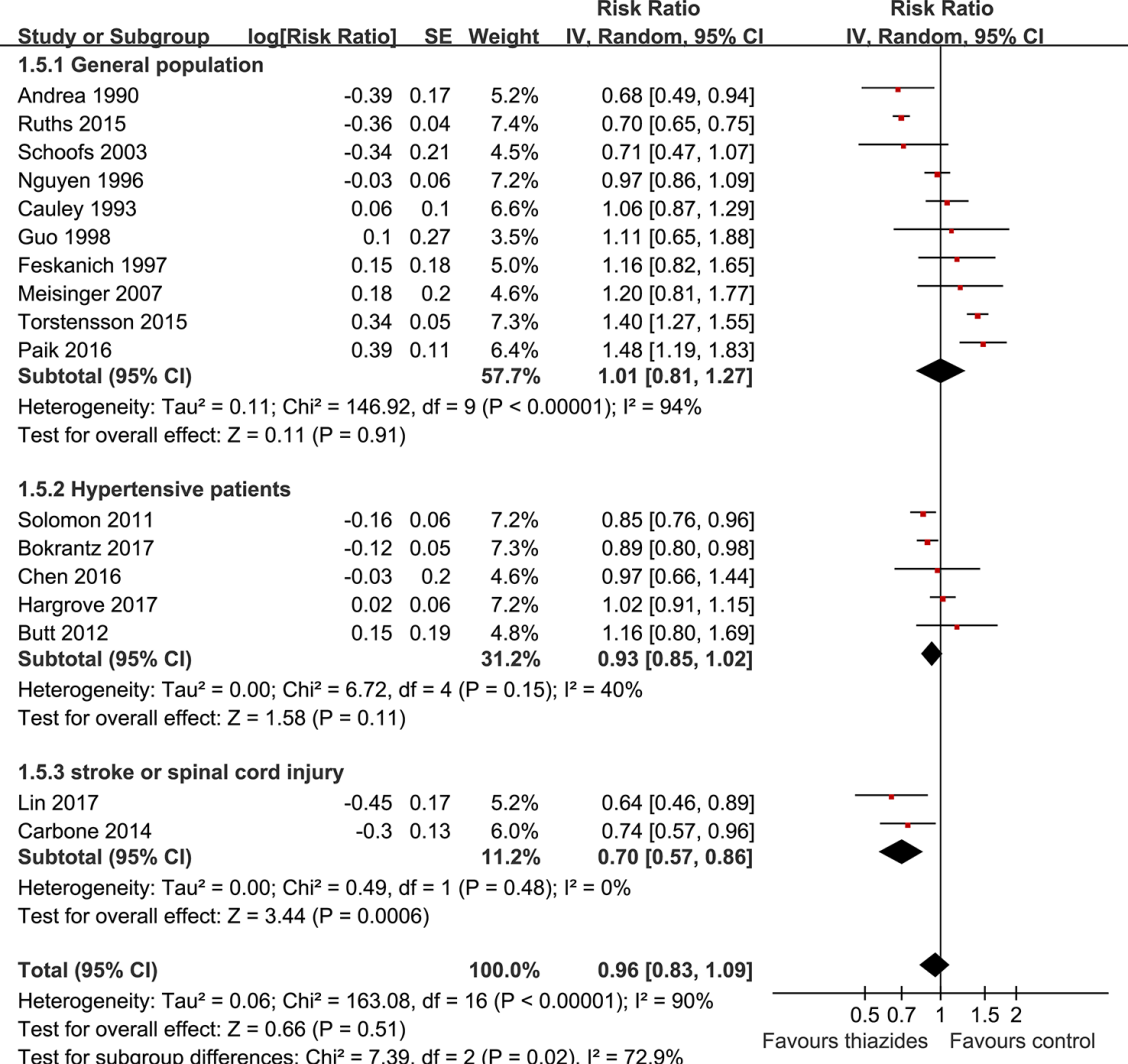
Subgroup analyses for the association between the use of thiazide diuretics and the incidence of fracture by sources of the participants.

The potential influences of other study characteristics on the association between thiazide diuretics and the risk of osteoporotic fracture were presented in **Table 3**. We found that use of thiazide diuretics at baseline did not significantly affect the risk of osteoporotic fracture regardless of study characteristics, such as study design, ethnicity, sample size, strategies for ascertainment of thiazides use, and extent of variables adjustment when presenting the results. Interestingly, we found that the sources of the participants may be an important determinant for the association between thiazide diuretics and subsequent risk of osteoporotic fracture (**Figure 3**). In detail, use of thiazide diuretics was associated with significantly reduced risk of fracture in patients with new-onset stroke or spinal cord injury (RR: 0.70, 95% CI: 0.57 to 0.86, p < 0.001; **Figure 3**), but not in those of community-dwelling population or hypertensive patients (p for subgroup difference = 0.02; **Figure 3**). Moreover, use of thiazide diuretics was associated with reduced risk of hip fracture (RR: 0.81, 95% CI: 0.69 to 0.94, p = 0.006), but not arm fracture. In addition, a significant association between use of thiazide diuretics and reduced fracture risk was observed in studies with follow-up duration ≤ 5 years (RR: 0.89, 95% CI: 0.80 to 1.00, p = 0.04), but not in those with follow-up duration > 5 years. However, the influences of the site of fracture and the follow-up durations on the association between thiazide diuretics and incidence of osteoporotic fracture were not significant (p values for subgroup difference = 0.24 and 0.43, respectively).

**Table 3:** Subgroup analysis for the association between thiazides use and the risk of fracture.

Variables	Dataset number	RR (95% CI)	P for subgroup effect	I^2^	P for subgroup difference
**Study design**					
PC	9	1.00 [0.86, 1.16]	0.99	72%	
RC	8	0.91 [0.73, 1.14]	0.42	95%	0.51
**Sex**					
Male	4	0.78 [0.63, 0.96]	0.02	80%	
Female	5	1.02 [0.79, 1.30]	0.89	89%	0.11
**Ethnicity**					
Asians	2	0.78 [0.51, 1.17]	0.23	61%	
Non-Asians	15	0.98 [0.85, 1.13]	0.75	91%	0.30
**Number of participants**					
≥10,000	9	0.88 [0.76, 1.01]	0.07	54%	
<10,000	8	1.04 [0.84, 1.28]	0.72	95%	0.19
**Participants characteristics**					
General population	10	1.01 [0.81, 1.27]	0.91	94%	
Hypertensive	5	0.93 [0.85, 1.02]	0.11	40%	
Stroke or spinal cord injury	2	0.70 [0.57, 0.86]	< 0.001	0%	0.02
**Follow-up**					
≤5 years	8	0.89 [0.80, 1.00]	0.04	62%	
>5 years	9	1.04 [0.82, 1.32]	0.74	94%	0.24
**Site of fracture**					
Hip fracture	9	0.81 [0.69, 0.94]	0.006	59%	
Arm fracture	3	0.92 [0.70, 1.21]	0.55	59%	0.43
**Ascertainment of thiazides use**					
Self-report	7	1.07 [0.90, 1.27]	0.44	68%	
Prescription records	10	0.89 [0.74, 1.07]	0.21	93%	0.15
**Numbers of variables adjusted**					
≤3	4	0.94 [0.72, 1.23]	0.67	90%	
>3	13	0.96 [0.83, 1.11]	0.58	87%	0.91

CI, confidence interval; PC, prospective cohort; RC, retrospective cohort; RR, risk ratio.

### Publication Bias

**Figure 4:**
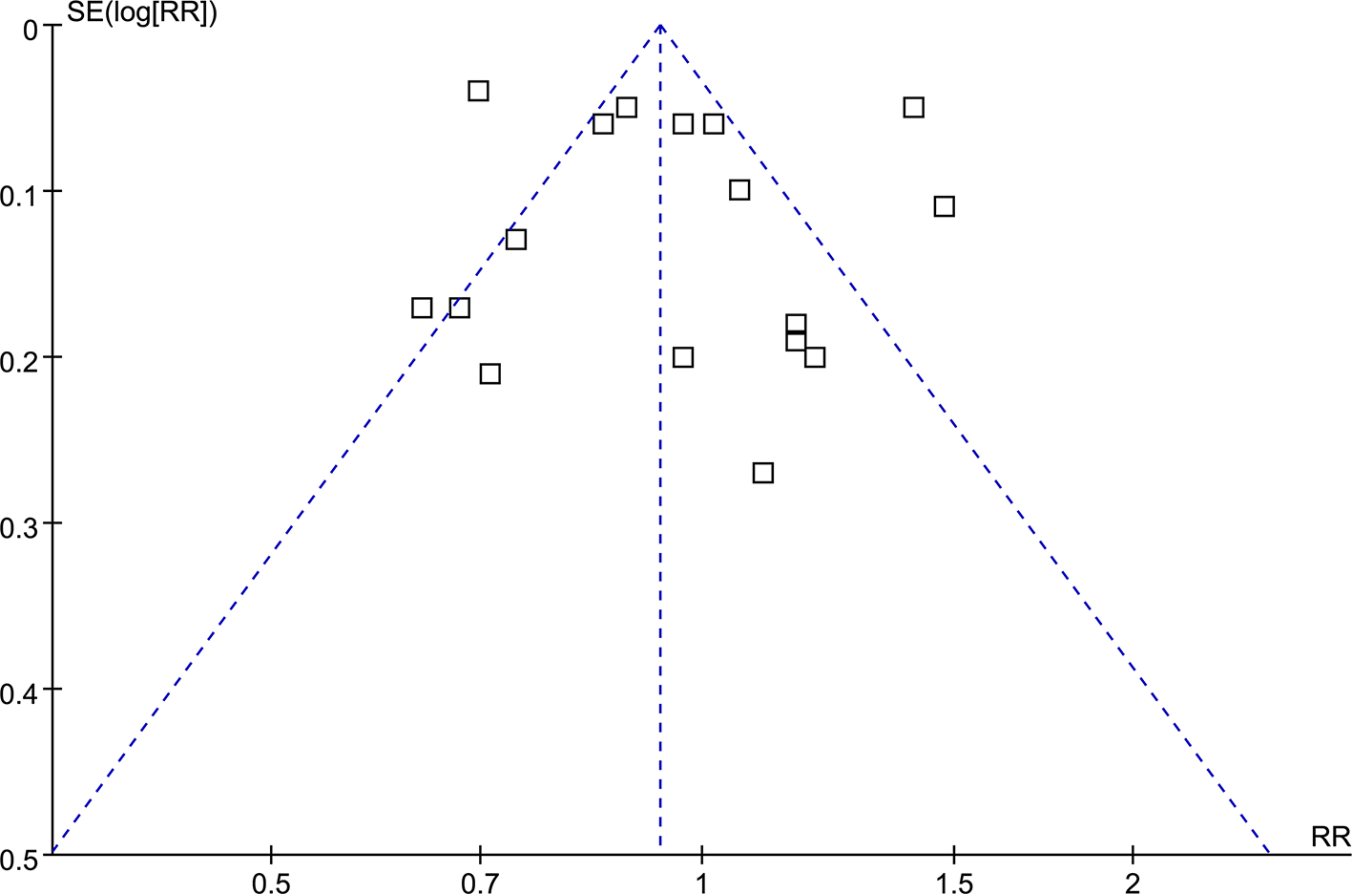
Funnel plots for the meta-analysis of the association between the use of thiazide diuretics and the incidence of fracture.

The funnel plots regarding the association between use of thiazide diuretics at baseline and incidence of overall fracture were shown in **Figure 4**. The funnel plots were symmetrical on visual inspection, suggesting low chance of significant publication bias. Results of Egger’s regression test also suggested that no significant publication bias (p = 0.55).

## Discussion

In this meta-analysis of cohort studies, we found that use of thiazide diuretics are not associated with significantly affected risk of osteoporotic fracture. The robustness of the study was further confirmed by the results of sensitivity analyses. Moreover, we found that the source and characteristics of the patients may be an important determinant for the association between use of thiazide diuretics and risk of osteoporotic fracture. Use of thiazide diuretics is associated with reduced risk of osteoporotic fracture in patients of acute clinical status including those with new-onset stroke or spinal cord injury, but not in those with generally good status such as community-dwelling population or hypertensive patients. These results challenged findings of previous studies that thiazide diuretics may be preventative against the incidence of osteoporotic fracture because of their efficacy for lowering urinary excretion of calcium.

The biological basis for the hypothesized association between use of thiazide diuretics and reduced risk of osteoporotic fracture are the potential benefits of thiazide diuretics on surrogate outcomes including urinary excretion of calcium and BMD (Laroche and Mazieres, 1998; Lacroix et al., 2000). However, this is challenged by a recent meta-analysis which failed to show that thiazides improved BMD (Cheng et al., 2018). Moreover, recent evidence suggests that strategies that favorably affect calcium homeostasis or BMD of the body may not necessarily translate to beneficial influence on clinical outcomes such as osteoporotic fracture. In a recently published meta-analysis, supplementation of calcium or/and vitamin D, previously established cornerstone therapy for osteoporosis, was shown to be not associated with a lower risk of fractures among community-dwelling people over 65 years (Zhao et al., 2017). On the other hand, a previous RCT indicated that supplementation of calcium or/and vitamin D lowered the risk of osteoporotic fracture in individuals living in residential institutions (Chapuy et al., 1992). Interestingly, results of our subgroup analyses also suggested that the source and characteristics of the participants may be important determinant for the association between use of thiazide diuretics and the risk of osteoporotic fracture incidence. We did find a significant association between use of thiazide diuretics and the risk of osteoporotic fracture in patients with acute clinical status including new-onset stroke or spinal cord injury, but did not in community-dwelling participants or hypertensive patients. Taken together, these results suggested that benefits of certain treatment strategies on BMD may not translate to favorable influence on fracture risk in participants with relatively good condition such as community-dwelling population or hypertensive patients. In contrast, beneficial efficacy of certain treatment strategies on BMD may translate to lowering effectiveness on fracture risk in individuals of poor clinical status, such as those with poor mobility, infrequent sun exposure, and poor diet without sufficient intake of calcium and vitamin D, probably because these patients are more likely to have osteoporosis. Therefore, the potential association between thiazide diuretics and risk of osteoporotic fracture may be different among community-dwelling population, hypertensive patients, and patients with acute clinical status including new-onset stroke or spinal cord injury.

It should be also noted that despite of the potential benefits of thiazide diuretics on urinary excretion of calcium and BMD, use of thiazide diuretics may also be related to factors which may expose the participants to increased risk of osteoporotic fracture. For example, long-term use of thiazide diuretics may induce hyponatremia (Glover and Clayton, 2012), which has recently been recognized as a potential risk factor for osteoporotic fracture (Upala and Sanguankeo, 2016; Ayus et al., 2017). Moreover, prevalence of orthostatic hypotension was reported to be as high as 65% in elderly patients who took thiazide diuretics (Scalco et al., 2000), and these patients were at higher risk for falls (Hartog et al., 2017), a common cause of osteoporotic fracture. It is possible that hyponatremia, orthostatic hypotension, and falls resulted by the use of thiazide diuretics may offset their favorable influences on calcium homeostasis and BMD, which may finally lead to an insignificant influence of thiazide diuretics on the risk of osteoporotic fracture.

Although insignificant, results of our subgroup analyses also suggested that sex of the individuals, follow-up durations, and site of fractures may influence the association between use of thiazide diuretics and the risk of osteoporotic fracture. We found use of thiazide diuretics was associated with reduced risk of osteoporotic fracture in males rather than females, in short-term studies rather than long-term ones, and in those reporting outcomes of hip fractures rather than arm fractures. These results should be interpreted cautiously because the differences between the subgroups were not statistically significant. Future well-designed cohort studies with adequate sample size are needed to explore whether the above study characteristics (sex of the participants, follow-up durations, and sites of fractures) have significant influence on the association between use of thiazide diuretics and the risk of osteoporotic fracture.

Our study has limitations which should be considered when interpreting the results. Firstly, significant heterogeneity exists among the included studies. Although we explored the potential source of heterogeneity by performing the subgroup analyses, these results should be interpreted with caution because limited numbers of studies were available for each stratum of subgroups. Moreover, we did not have access to individual-patient based data, and the subgroup analyses were performed on the basis of study-level results. The potential influence of study characteristics on the outcome should be confirmed in future large-scale cohort studies. Secondly, as inherited in the meta-analysis of observational studies, we could not exclude the possibility of other factors that may confound the association between use of thiazide diuretics and the risk of osteoporotic fracture despite that we pooled the result with the most adequately adjusted data. Thirdly, we were unable to evaluate whether the association between use of thiazide diuretics and the risk of osteoporotic fracture differed according to the doses and durations of thiazides treatment. Finally, whether the association between use of thiazide diuretics and the risk of osteoporotic fracture differed among individual medication of thiazide diuretics should also be determined.

## Conclusions

In conclusion, results of our meta-analysis indicated that use of thiazide diuretics is not associated with significantly affected risk of overall osteoporotic fracture. However, the association between use of thiazide diuretics and fracture risk may differ according to the general status of the participants.

## Data Availability Statement

The raw data supporting the conclusions of this manuscript will be made available by the authors, without undue reservation, to any qualified researcher.

## Author Contributions

JW conceived and designed the study. JW and KS selected the studies and collected the data. JW, LL, WS and SM analyzed data, and all authors interpreted the results. JW drafted and revised the paper. KS, LL, WS and SM revised the draft paper. All authors read and approved the final version of the manuscript.

## Conflict of Interest

The authors declare that the research was conducted in the absence of any commercial or financial relationships that could be construed as a potential conflict of interest.
